# Eicosapentaenoic Acid Influences the Lipid Profile of an In Vitro Psoriatic Skin Model Produced with T Cells

**DOI:** 10.3390/biom13091413

**Published:** 2023-09-19

**Authors:** Sophie Morin, Andréa Tremblay, Elizabeth Dumais, Pierre Julien, Nicolas Flamand, Roxane Pouliot

**Affiliations:** 1 Centre de Recherche en Organogénèse Expérimentale de l’Université Laval/LOEX, Axe Médecine Régénératrice, Centre de Recherche du CHU de Québec-Université Laval, 1401 18e Rue, Québec, QC G1J 2Z4, Canada; sophie.morin.7@ulaval.ca (S.M.); andrea.tremblay.4@ulaval.ca (A.T.); 2 Faculté de Pharmacie, Université Laval, Québec, QC G1V 0A6, Canada; 3 Centre de Recherche de l’Institut Universitaire de Cardiologie et de Pneumologie de Québec, Québec, QC G1V 4G5, Canada; elizabeth.dumais.1@ulaval.ca (E.D.); nicolas.flamand@criucpq.ulaval.ca (N.F.); 4 Canada Excellence Research Chair on the Microbiome-Endocannabinoidome Axis in Metabolic Health (CERC-MEND), Université Laval, Québec, QC G1V 0A6, Canada; 5 Centre de Recherche du CHU de Québec-Université Laval, Axe Endocrinologie et Néphrologie, Université Laval, Québec, QC G1V 4G2, Canada; pierre.julien@crchudequebec.ulaval.ca; 6 Département de Médecine, Faculté de Médecine, Université Laval, Québec, QC G1V 0A6, Canada

**Keywords:** psoriasis, T cells, n-3 PUFAs, bioactive lipid mediators

## Abstract

Psoriasis is a skin disease characterized by epidermal hyperplasia and an inappropriate activation of the adaptive immunity. A dysregulation of the skin’s lipid mediators is reported in the disease with a predominance of the inflammatory cascade derived from n-6 polyunsaturated fatty acids (n-6 PUFAs). Bioactive lipid mediators derived from arachidonic acid (AA) are involved in the inflammatory functions of T cells in psoriasis, whereas n-3 PUFAs’ derivatives are anti-inflammatory metabolites. Here, we sought to evaluate the influence of a supplementation of the culture media with eicosapentaenoic acid (EPA) on the lipid profile of a psoriatic skin model produced with polarized T cells. Healthy and psoriatic skin substitutes were produced following the auto-assembly technique. Psoriatic skin substitutes produced with or without T cells presented increased epidermal and dermal linolenic acid (LA) and AA levels. N-6 PUFA lipid mediators were strongly measured in psoriatic substitutes, namely, 13-hydroxyoctadecadienoic acid (13-HODE), prostaglandin E_2_ (PGE_2_) and 12-hydroxyeicosatetraenoic acid (12-HETE). The added EPA elevated the amounts of EPA, n-3 docosapentaenoic acid (DPA) and docosahexaenoic acid (DHA) in the epidermal and dermal phospholipids. The EPA supplementation balanced the production of epidermal lipid mediators, with an increase in prostaglandin E_3_ (PGE_3_), 12-hydroxyeicosapentaenoic acid (12-HEPE) and *N*-eicosapentaenoyl-ethanolamine (EPEA) levels. These findings show that EPA modulates the lipid composition of psoriatic skin substitutes by encouraging the return to a cutaneous homeostatic state.

## 1. Introduction

Psoriasis is a complex autoimmune and multifactorial skin disease affecting about 3% of the global population [1,2]. Psoriatic skin is characterized by an exaggerated epidermal proliferation of keratinocytes, leading to thickening of the skin. The process of keratinocyte differentiation is also affected, with incomplete cell differentiation causing the corneocytes of the stratum corneum to retain their nuclei, a phenomenon also called parakeratosis [3]. Increased angiogenesis is also reported in psoriatic plaques [4]. Another characteristic of the pathology involves an aberrant activation of the immune system, leading to leukocyte infiltration into psoriatic skin. The activated T cells release inflammatory cytokines that participate in the abnormal differentiation and the altered expression of many epidermal proteins, including increased expression of early differentiation keratins [5]. In fact, the dysregulation of immune cells in psoriasis, and particularly the alteration of T cells, plays a critical role in the development and the maintenance of psoriatic plaques. It is widely accepted that the lesional skin and peripheral blood of psoriatic patients display increased numbers of CD4 helper T cells (Th cells) of type 1 and type 17 (Th1 and Th17 cells). Together, these cells secrete large amounts of cytokines and chemokines such as TNF-α, IFN-γ, IL-17A and IL-6, all participating in the inflammatory loop generating and perpetuating the psoriatic lesions [6]. These leukocytes also interact with psoriatic keratinocytes to generate the complete inflammatory environment of the disease [7].

Psoriasis is also accompanied by a significant dysregulation of the cutaneous lipid portrait with an increase in n-6 polyunsaturated fatty acid (n-6 PUFA) metabolism. Thus, high levels of inflammatory lipid mediators derived from n-6 PUFAs are reported in psoriatic plaques such as prostaglandin E_2_ (PGE_2_), leukotriene B_4_ (LTB_4_), 9-hydroxyoctadecadienoic acid (9-HODE) and 12-hydroxyeicosatetraenoic acid (12-HETE) [8]. These bioactive lipid mediators play multiple roles in the regulation of leukocyte functions, particularly T cell activity. In fact, the first evidence of the role of lipid mediators on T cell functions was reported in the early 2000s with studies showing the ability of LTB_4_ to recruit T cells into inflammatory sites such as psoriatic plaques [9,10,11,12]. LTB_4_ regulates leukocyte recruitment in injured skin, and 12-HETE induces the chemotaxis of neutrophils in psoriatic lesions [13,14]. The effects of endocannabinoid derivatives on T cells, such as *N*-acylethanolamines (NAEs) and monoacylglycerols (MAGs), are less well known [15]. Unlike n-6 PUFAs, n-3 PUFAs are regarded as being a source of anti-inflammatory metabolites, possessing strong therapeutic potential in numerous diseases [16]. In the last decade, a myriad of clinical trials have evaluated the beneficial effects of oral supplementation with n-3 PUFAs on psoriasis achieved by shifting the balance between the n-3 and n-6 families in favor of n-3 PUFAs [17,18,19]. While some studies have shown beneficial effects of n-3 PUFAs on certain symptoms of psoriasis (including disease severity and the psoriasis area and severity index (PASI) score), others were unsuccessful in demonstrating significant clinical improvements [20,21].

There is thus a flagrant lack of consensus regarding the role and concrete actions of n-3 PUFAs in psoriasis. In the past, different studies carried out by our team have assessed the impact of n-3 PUFAs, including alpha-linolenic acid (ALA) and docosahexaenoic acid (DHA), on the main characteristics of psoriasis using our tissue-engineered psoriatic skin model, and we have demonstrated that both of them attenuate the psoriatic characteristics of the model [22,23,24]. These experiments also showed a strong incorporation of n-3 PUFAs in the membrane phospholipids of the substitutes as well as the metabolization of ALA and DHA into EPA [22,24]. We then extended these studies to a more complex model that included T cells, in order to determine the potential action of n-3 PUFAs on the function of these immune cells in a psoriatic context. These subsequent experiments also exhibited a high rate of conversion of ALA into EPA in the epidermal phospholipids of the supplemented substitutes [23]. Since these results demonstrated such a high metabolization of the added n-3 PUFAs to EPA, we hypothesized that the antipsoriatic effects of n-3 PUFAs might be directly mediated by EPA itself. Therefore, we then demonstrated the potential of EPA to decrease T-cell mediated psoriatic hallmarks [25]. However, the lipid profile of the T-cell enriched psoriatic skin model has never been characterized, nor the impact of EPA supplementation on the lipid characteristics of psoriasis has been revealed. Therefore, the present study was intended to evaluate the influence of EPA on the lipid profile of psoriatic skin substitutes produced with polarized T cells, mainly by measuring its impact on the phospholipid composition of the epidermis and dermis of the psoriatic substitutes, as well as the production levels of bioactive lipid mediators derived from both n-3 and n-6 PUFAs. The present study also describes the effects of the addition of polarized T cells on the lipid components of the psoriatic model.

## 2. Materials and Methods

### 2.1. Skin Cell Culture

The study was conducted following the standard procedures and approved by the Research Ethics Committee of the CHU de Québec-Université Laval. In addition, all donors signed a consent document in agreement with the Declaration of Helsinki. Fibroblasts and keratinocytes from healthy donors were extracted from the breast reduction skin biopsies of three Caucasian women aged, 18, 38 and 46 years old. Fibroblasts and keratinocytes obtained from psoriatic donors were extracted from punch biopsies of three plaque psoriatic patients aged 36 (woman, back biopsy, 5–10% psoriasis extent no treatment), 39 (man, PASI score of 17, no treatment known) and 64 (woman, back biopsy, 20% psoriasis extent, no treatment) years old. The epithelial cells were extracted and frozen before use, as described elsewhere [23,24,26]. Human fibroblasts were cultured in the Dulbecco–Vogt modification of Eagle’s medium (DMEM) (Gibco, Life Technologies, New York, NY, USA) with a supplementation of 10% bovine growth serum (FB Essence, Seradigm, Mississauga, ON, Canada), 100 UI/mL penicillin G (Sigma, Oakville, ON, Canada) and 25 μg/mL gentamicin (Gemini Bio-Products, Sacramento, CA, USA). The dermal cells were supplemented with 50 μg/mL ascorbic acid (Sigma, Oakville, ON, Canada) to allow the formation of the extracellular matrix. Human keratinocytes were cultured in DME mixed with Ham’s F12 medium (3:1) (DMEH) (Gibco, Life Technologies, New York, NY, USA) with the addition of 5% FetalClone II serum (Galenova, Saint-Hyacinthe, QC, Canada), 5 μg/mL insulin (Sigma, Oakville, ON, Canada), 0.4 μg/mL hydrocortisone (Galenova, St-Hyacinthe, QC, Canada), 10^−10^ M cholera toxin (Sigma, Oakville, ON, Canada), 10 ng/mL human epidermal growth factor (EGF) (Ango. Inc., San Ramon, CA, USA), 60 μg/mL penicillin, 25 μg/mL gentamicin and 50 μg/mL ascorbic acid.

Skin substitutes were produced in unsupplemented media for healthy substitutes (HS), psoriatic substitutes (PS) and psoriatic substitutes produced with T cells (PS^+T^) or in culture media supplemented with 10 μM EPA for psoriatic substitutes supplemented with EPA (PS^+EPA^) and psoriatic substitutes produced with T cells and supplemented with EPA (PS^+T+EPA^). A corresponding volume of ethanol, similar to the volume of n-3 PUFA used, was added to the unsupplemented media. For the n-3 PUFA supplementation, a solution was prepared by dissolving EPA (Cedarlane, Burlington, ON, Canada) in 99% ethanol (Greenfield Global, Brampton, ON, Canada) as explained elsewhere [23]. The EPA solution was added directly to the culture media, which contained antioxidants and bovine serum albumin (BSA), in order to obtain a final concentration of 10 μM EPA. Human fibroblasts and keratinocytes were both maintained under these conditions: 37 °C and 8% CO_2_.

### 2.2. Production of Healthy and Psoriatic Skin Substitutes

Each skin substitute was reconstructed according to the self-assembly method described previously elsewhere [27]. Fibroblasts at passage 5 were cultured over a period of 28 days in 6-well plates with a paper anchor, at a concentration of 1.2 × 10^4^ cells/well. Ascorbic acid was added to the medium to form fully manipulable dermal sheets. Human T cells were then isolated from the blood extract of healthy donors and polarized and activated according to the protocol previously reported by our team [27]. Briefly, T cells were isolated by negative selection from whole blood. Once isolated, T cells were polarized for 3 days towards Th1 and Il-17A phenotypes using specific antibody cocktails [27]. T cells were activated using a mixture of phorbol-12-myristate 13-acetate (PMA) and ionomycin (Sigma, St. Louis, MO, USA) and kept in culture for 4 more days with the addition of IL-2 and IL-23 (30 U/mL for IL-2 and 20 ng/mL for IL-23, R&D Systems, Burlington, ON, Canada). Human keratinocytes at passage 2 were then seeded onto one fibroblast sheet out of two (1.2 × 10^6^ cells/well), and T cells were seeded onto the other fibroblast sheet (0.5 × 10^6^ cells/well at an equal ratio of Th1 to Th17 cells). Keratinocytes and T cells were separately co-cultured with fibroblasts in submerged conditions for 7 days and culture media were changed every day for keratinocytes, and every 3 days for T cells. Then, the two dermal sheets were stacked, with the dermal sheet with T cells under the one containing keratinocytes. The same day, the skin assemblies were raised to the air–liquid interface and cultured for three additional weeks with the addition of cytokines to ensure T cell survival (10 U/mL IL-2 and 20 ng/mL IL-23) [27]. Once the cell culture was completed, the dermis and epidermis of the skin substitutes were separated using forceps, and each skin layer (the epidermal keratinocyte layer and the dermal fibroblast layer) was frozen separately until needed.

### 2.3. Histology

The reconstructed skin substitutes were fixed in formol (ThermoFisher Scientific, Waltman, MA, USA) and then included in paraffin. For staining with hematoxylin and eosin dyes (H&E), 6 μm thick sections were mounted on slides. A total of 6 skin substitutes were analyzed (N = 3 donors; n = 2 skin substitute per donor).

### 2.4. Immunofluorescence

For each skin substitute condition, 6 μm thick slices were fixed in acetone for 10 min at −20 °C. Each slice was then covered with the primary antibodies, diluted in phosphate-buffered saline (PBS) containing 1% BSA, for 45 min in a dark humidified chamber. The primary antibodies used were keratin 17 (K17, Abcam, ab51056, Cambridge, UK), keratin 14 (K14, Cedarlane, CLPRB-155B, Burlington, ON, Canada) and keratin 10 (K10, Abcam, ab9025, Cambridge, UK). After 3 rinses with PBS, each slice was incubated with the secondary antibodies, diluted in PBS 1% BSA, for 30 min in a dark humidified chamber. The secondary antibodies used were anti-mouse Alexa 488 and anti-rabbit Alexa 488 (Life Technologies, A11001 (mouse) and A21206 (rabbit), Carlsbad, CA, USA). Each skin substitute was placed in a mounting medium containing 4′-6′-diamidino-2-phenylindole (DAPI) (Fluoromount-G, SouthernBiotech, Birmingham, AL, USA), which stained the cell nuclei. Finally, images of each skin substitute were taken with a Zeiss microscope fitted with an AxioCam HR Rev3 camera (Oberkochen, Germany).

### 2.5. Analysis of Epidermal and Dermal Phospholipids

For the analysis of skin phospholipids, a gas chromatograph with a flame ionization detector (GC-FID) was used as described elsewhere [24,28,29]. In summary, the dermal and epidermal compartments of the skin substitutes (previously frozen separately after cell culture) were incubated in a mixture of chloroform and methanol (2:1 vol/vol) in order to extract the lipids (a technique modified from the Folch method). Gas chromatography was performed using a HP5890 gas chromatograph (Hewlett-Packard, Toronto, ON, Canada) with an HP-88 capillary column (Agilent Technologies, Santa Clara, CA, USA) coupled with a flame ionization detector.

### 2.6. Analysis of Lipid Mediators by LC-MS/MS

For the analysis of skin lipid mediators, the epidermal skin compartment (previously frozen after cell culture) was first reduced to powder using a Cryomill grinder (Cryomill MM400; Retsch^®^, Newtown, PA, USA) as described elsewhere [22,23,24]. The ground epidermis was then suspended in 50 mM Tris hydrochloride and denatured in methanol. The lipids were extracted as described before and reconstituted in 50 μL of a liquid–chloroform solvent (50/50) [30]. Finally, 40 μL was injected onto a HPLC column (Kinetex C8, 150 × 2.1 mm, 2.6 μm; Phenomenex, Torrance, CA, USA) and analyzed using LC-MS/MS [29,30,31]. The bioactive lipid mediators were then quantified using the appropriate deuterated standards.

### 2.7. Statistics

Prism9 software (Graphpad Software, La Jolla, CA, USA) was used for all the statistical analyses of the study. The results in the present study are expressed as mean ± standard deviation (SD), and the tests used for the statistical analyses were analyses of variance (ANOVAs) followed by Tukey’s or Bonferroni’s post hoc test. The threshold for statistical significance was set at *p*-values of < 0.05.

## 3. Results

### 3.1. EPA Ameliorates Skin Physiology of Psoriatic Skin Substitutes

**Figure 1:**
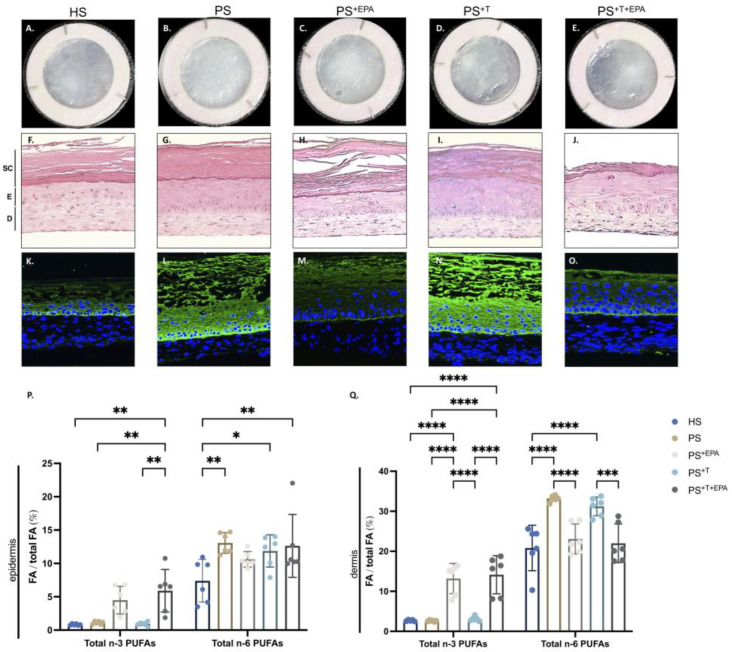
Histology, immunofluorescence and total phospholipid quantification of the skin substitutes. (**A**–**E**) Macroscopic representations of healthy and psoriatic skin substitutes. (**F**–**J**) Histological representations of H&E staining of healthy and psoriatic skin substitutes. The scale bar represents 100 μm. (**K**–**O**) Indirect immunofluorescence staining of K14 expression in healthy and psoriatic skin substitutes. K14 expression is shown in green. The cell nuclei were counterstained with DAPI reagent and are shown in blue. The dashed white lines represent the basement membrane. (**P**) Characterization of epidermal total fatty acids using gas chromatography. Impact of EPA supplementation on total n-3 and n-6 PUFA percentages (n-3 and n-6 PUFAs/ total fatty acids). (**Q**) Characterization of dermal total fatty acids using gas chromatography. Impact of EPA supplementation on total n-3 and n-6 PUFA percentages (n-3 and n-6 PUFAs/ total fatty acids). The values are presented as percentages (N = 3 donors, n = 2 skin substitutes per donor). Statistical significance was determined using two-way ANOVA followed by Tukey’s post hoc test. Significant differences are indicated by asterisks (* *p* < 0.05; ** *p* < 0.01; *** *p* < 0.001; **** *p* < 0.0001). Abbreviations: HS: healthy substitutes; EPA: eicosapentaenoic acid; PS: psoriatic substitutes; PS^+EPA^: psoriatic substitutes supplemented with EPA; PS^+T^: psoriatic substitutes produced with T cells; PS^+T+EPA^: psoriatic substitutes produced with T cells and supplemented with EPA; and T: T cells.

Healthy and psoriatic skin substitutes were produced using tissue engineering with unsupplemented media (HS, PS and PS^+T^) or media supplemented with 10 μM EPA (PS^+EPA^ and PS^+T+EPA^). The upper surface of PS and PS^+T^ was thick and patchy as compared with HS, which was smooth and even (Figure 1A,B,D). The EPA supplementation in PS^+EPA^ and PS^+T+EPA^ improved the morphological appearance of the substitutes (Figure 1C,E). All skin substitute sections were stained with H&E to detail their histological features. The psoriatic substitutes (PS and PS^+T^) had a thicker living epidermis than HS, a classical hallmark of the pathology (Figure 1F,G,I). The substitutes supplemented with EPA exhibited an epidermal thickness that approximated that of the healthy condition (Figure 1H,J). Immunofluorescence staining was performed to determine the expression of K14 in the skin substitutes, a protein found in the basal keratinocytes of the skin and overexpressed in psoriasis as seen in PS and PS^+T^ (Figure 1L,N) [32]. The skin substitutes supplemented with EPA were closer to the level of K14 expression found in HS, implying a possible return to skin homeostasis (Figure 1M, O). The incorporation of EPA into the phospholipid fractions of the epidermis and dermis was then evaluated using GC-FID. Higher amounts of total n-3 PUFAs were quantified both in the epidermis and dermis membrane phospholipids following EPA supplementation. This was observed as much in PS^+EPA^ as in PS^+T+EPA^ (Figure 1P, Q). Increased amounts of total n-6 PUFAs were observed in psoriatic substitutes with or without T cells (PS and PS^+T^) compared with their healthy counterpart (HS), but the addition of EPA did not seem to affect the n-6 PUFA percentages (Figure 1P). However, important differences were observed in the quantities of dermal phospholipids with significant decreases in substitutes supplemented with EPA (PS^+EPA^ and PS^+T+EPA^) compared with their equivalents PS and PS^+T^ (Figure 1Q). The results of total n-3 and n-6 PUFA quantities (μg per g of tissue) are presented in the supplementary material (see Supplementary Figure S1).

### 3.2. Incorporation of n-3 PUFA into the Phospholipid Fraction of the Epidermis and Dermis of the Skin Substitutes

**Figure 2:**
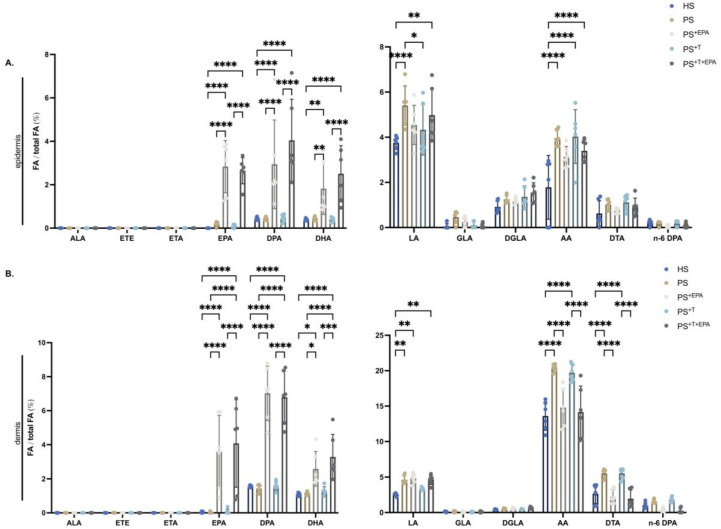
PUFA incorporation into epidermal and dermal phospholipids of the skin substitutes. Characterization of the levels of n-3 and n-6 PUFA amounts in the phospholipids of the epidermis and dermis of HS, PS, PS^+EPA^, PS^+T^ and PS^+T+EPA^; (**A**) n-3 and n-6 PUFA levels in the epidermal phospholipid fraction of the skin substitutes; (**B**) n-3 and n-6 PUFA levels in the dermal phospholipid fraction of the skin substitutes. PUFAs were quantified using gas chromatography, and results are presented as percentages (fatty acids/total fatty acids) (N = 3 donors; n = 2 skin substitutes per donor). Statistical significance was determined using two-way ANOVA followed by Tukey’s post hoc test. Significant differences are indicated by asterisks (* *p* < 0.05; ** *p* < 0.01; *** *p* < 0.001; **** *p* < 0.0001). Abbreviations: AA: arachidonic acid; ALA: alpha-linolenic acid; DGLA: dihomo-γ-linolenic acid; DHA: docosahexaenoic acid; DPA: docosapentaenoic acid; DTA: docosatetraenoic acid; EPA: eicosapentaenoic acid; ETA: eicosatetraenoic acid; ETE: eicosatrienoic acid; GLA: γ-linolenic acid; HS: healthy substitutes; LA: linoleic acid; T: T cells; n-3: omega-3; n-6: omega-6; PUFAs: polyunsaturated fatty acids; PS: psoriatic substitutes; PS^+EPA^: psoriatic substitutes supplemented with EPA; PS^+T^: psoriatic substitutes produced with T cells; and PS^+T+EPA^: psoriatic substitutes produced with T cells and supplemented with EPA.

N-3 and n-6 PUFA metabolization in the dermis and epidermis of the skin substitutes was evaluated by using a GC-FID. In the epidermis, significantly higher quantities of EPA, n-3 DPA and DHA were measured in the phospholipid fraction of PS^+EPA^ as compared with its counterpart PS (+8.0-fold, +6.6-fold and +4.2-fold, respectively) (Figure 2A). Similar results were collected for skin substitutes produced with T cells: the amounts of EPA, n-3 DPA and DHA were significantly upregulated in the epidermal phospholipids of PS^+T+EPA^ compared with PS^+T^ (+33.0-fold, +8.8-fold and +4.7-fold, respectively) (Figure 2A). Analogous data were obtained for the dermis, with increased levels of long-chain n-3 PUFAs following EPA supplementation in PS^+EPA^ and PS^+T+EPA^ in contrast with unsupplemented substitutes (EPA: +196.0-fold in PS^+EPA^ and +44.3-fold in PS^+T+EPA^; n-3 DPA: +4.9-fold in PS^+EPA^ and +4.1-fold in PS^+T+EPA^; DHA: +2.2-fold in PS^+EPA^ and +2.6-fold in PS^+T+EPA^) (Figure 2B). Linoleic acid (LA) is the most abundant n-6 PUFA in the skin and its levels are further increased in psoriatic skin, as found here in the epidermis and dermis of PS compared with HS (Figure 2A,B) [29,33]. Significantly higher quantities of AA were measured in psoriatic substitutes with or without T cells (PS and PS^+T^) compared with HS, both in the epidermis and dermis which is representative of the disease (Figure 2A,B). The exogenous added EPA that was incorporated in the epidermis of PS^+EPA^ and PS^+T+EPA^ did not significantly alter the levels of AA (Figure 2A). In contrast, the dermis from skin substitutes supplemented with EPA (PS^+EPA^ and PS^+T+EPA^) presented reduced quantities of AA and docosatetraenoic acid (DTA) compared with their respective controls PS and PS^+T^ (AA: 1.4-fold reductions in PS^+EPA^ and PS^+T+EPA^; DTA: 2.5-fold reduction in PS^+EPA^ and 2.9-fold reduction in PS^+T+EPA^) (Figure 2B).

### 3.3. Upregulation of the n-3 PUFA Lipid Mediators following Supplementation of the Psoriatic Skin Substitutes with EPA

**Figure 3:**
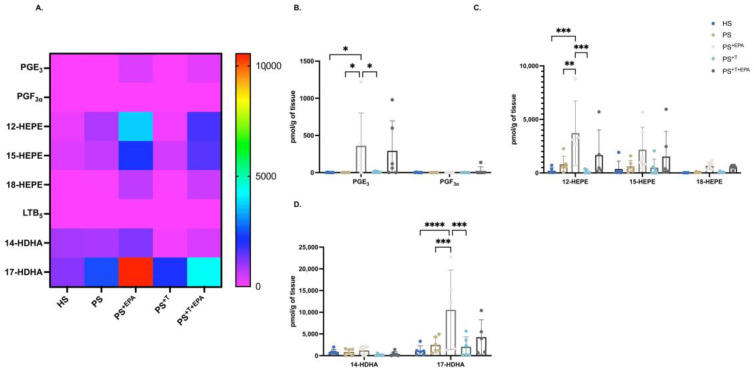
Impact of supplementation with EPA on the n-3 PUFA lipid mediator profile of the skin substitutes. Characterization of the levels of n-3-PUFA lipid mediators in the epidermis of HS, PS, PS^+EPA^, PS^+T^ and PS^+T+EPA^. (**A**) Heatmap of n-3-lipid mediators analyzed using LC-MS/MS in the epidermis of the skin substitutes. (**B**) Characterization of epidermal n-3 prostaglandins (PG). (**C**) Characterization of epidermal n-3 hydroxyeicosapentaenoic acids (HEPE). (**D**) Characterization of epidermal n-3-hydroxydocosahexaenoic acids (HDHA) (N = 3 donors; n = 2 skin substitutes per donor). Statistical significance was determined using two-way ANOVA followed by Bonferroni’s post hoc test. Significant differences are indicated by asterisks (* *p* < 0.05; ** *p* < 0.01; *** *p* < 0.001; **** *p* < 0.0001). Abbreviations DHA: docosahexaenoic acid; EPA: eicosapentaenoic acid; HEPE: hydroxyeicosapentaenoic acid; HDHA: hydroxydocosahexaenoic acid; HS: healthy substitutes; T: T cells; n-3: omega-3; n-6: omega-6; PG: prostaglandin; PUFAs: polyunsaturated fatty acids; PS: psoriatic substitutes; PS^+EPA^: psoriatic substitutes supplemented with EPA; PS^+T^: psoriatic substitutes produced with T cells; and PS^+T+EPA^: psoriatic substitutes produced with T cells and supplemented with EPA.

Characterization of the n-3-lipid mediator profiles of HS, PS, PS^+EPA^, PS^+T^ and PS^+T+EPA^ using LC-MS/MS revealed that large amounts of n-3-lipid mediators were produced in the epidermal compartment of the skin substitutes, even in the presence of T cells (Figure 3A). The levels of n-3-lipid mediators were generally similar in HS and PS (Figure 3A–D). However, supplementation of the culture media with EPA in PS^+EPA^ and PS^+T+EPA^ elevated the overall levels of n-3-lipid mediators compared with HS and PS (Figure 3A). More specifically, EPA supplementation significantly increased the levels of PGE_3_, 12-HEPE and 17 hydroxydocosahexaenoic acid (17-HDHA) in PS^+EPA^ compared with PS. Despite not being statistically significant, an upward trend in PGE_3_, 12-HEPE, 15-HEPE and 17-HDHA was observable in PS^+T+EPA^ as compared with PS^+T^. The addition of EPA to psoriatic skin substitutes produced with T cells (PS^+T+EPA^) failed to increase the level of n-3-lipid mediators to the same extent as in PS^+EPA^, suggesting an impact of T cells on the production of n-3-lipid mediators (Figure 3B–D). The differences in the levels of EPA-derived lipid mediators between psoriatic conditions are presented in the supplementary material (see Supplementary Figure S2).

### 3.4. EPA Modulates the n-6 PUFA Lipid Mediator Profile of Psoriatic Skin Substitutes

**Figure 4:**
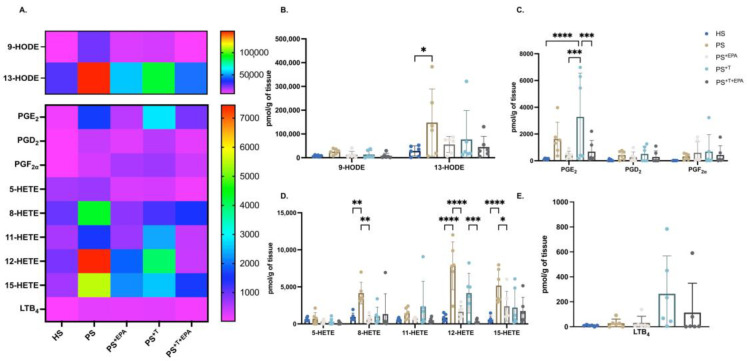
Impact of supplementation with EPA on the n-6-PUFA lipid mediator profile of the skin substitutes. Characterization of the levels of n-6-PUFA lipid mediators in the epidermis of HS, PS, PS^+EPA^, PS^+T^ and PS^+T+EPA^. (**A**) Heatmap of n-6-lipid mediators analyzed using LC-MS/MS in the epidermis of the skin substitutes. (**B**) Characterization of epidermal n-6 hydroxyocatadecadienoic acids (HODE). (**C**) Characterization of epidermal n-6 prostaglandins (PG). (**D**) Characterization of epidermal n-6-hydroxyeicosatetraenoic acids (HETE). Results are expressed as pmol per g of tissue. (**E**) Characterization of epidermal LTB_4_ levels (N = 3 donors; n = 2 skin substitutes per donor). Statistical significance was determined using two-way ANOVA followed by Bonferroni’s post hoc test. Significant differences are indicated by asterisks (* *p* < 0.05; ** *p* < 0.01; *** *p* < 0.001; **** *p* < 0.0001). Abbreviations: AA: arachidonic acid; HETE: hydroxyeicosatetraenoic acid; HODE: hydroxyoctadecadienoic acid; HS: healthy substitutes; LA: linoleic acid; T: T cells; n-3: omega-3; n-6: omega-6; PG: prostaglandin; PUFAs: polyunsaturated fatty acids; PS: psoriatic substitutes; PS^+EPA^: psoriatic substitutes supplemented with EPA; PS^+T^: psoriatic substitutes produced with T cells; and PS^+T+EPA^: psoriatic substitutes produced with T cells and supplemented with EPA.

Overall, the n-6 PUFA metabolites were transformed into their respective bioactive lipid derivatives in all skin substitutes. LA and AA lipid mediators were mostly detected in psoriatic skin substitutes as compared with HS (Figure 4A). An increase in the majority of the n-6 lipid mediators was detected in psoriatic substitutes produced with or without T cells (PS and PS^+T^) compared with HS, in particular for 13-HODE, PGE_2_, 8-HETE, 12-HETE and 15-HETE (Figure 4B–D). Significant increases in 13-HODE, 8-HETE, 12-HETE and 15-HETE levels were found in PS, while significantly increased levels of PGE_2_ were found in PS^+T^ (Figure 4B–D). After supplementation with EPA, the amounts of most n-6-lipid derivatives were lowered in PS^+EPA^ and PS^+T+EPA^. PGE_2_ was downregulated by the addition of EPA in PS^+T+EPA^ compared with PS^+T^ (Figure 4C). Among the hydroxy fatty acids (HFA), 12-HETE was the most affected mediator following supplementation with EPA, with significant decreases in its levels in both PS^+EPA^ and PS^+T+EPA^ (Figure 4D). 8-HETE and 15-HETE levels were also diminished in PS^+EPA^ compared with PS (Figure 4D). LTB_4_ was found mainly measured in psoriatic skin substitutes produced with T cells (PS^+T^ and PS^+T+EPA^) (Figure 4E). The differences in the levels of AA-derived lipid mediators between psoriatic conditions are presented in the supplementary material (see Supplementary Figure S2).

### 3.5. Modification of the NAE–Endocannabinoid Profile of the Psoriatic Skin Substitutes following EPA Supplementation

**Figure 5:**
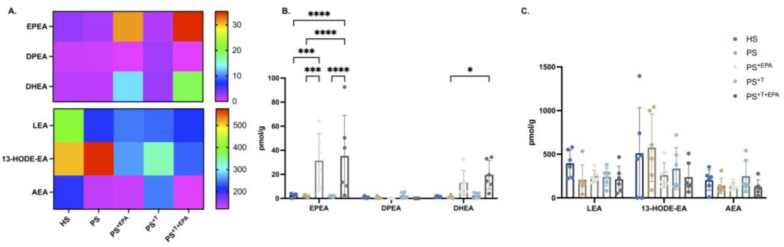
Impact of the supplementation with EPA on the NAE profile of the skin substitutes. Characterization of the NAE derivate levels of the epidermis of HS, PS, PS^+EPA^, PS^+T^ and PS^+T+EPA^. (**A**) Heatmap of the NAEs analyzed using LC-MS/MS in the epidermis of the skin substitutes. (**B**) Characterization of epidermal NAEs derived from n-3 PUFAs. (**C**) Characterization of epidermal NAEs derived from n-6 PUFAs. Results are expressed as pmol per g of tissue (N = 3 donors; n = 2 skin substitutes per donor). Statistical significance was determined using two-way ANOVA followed by Bonferroni’s post hoc test. Significant differences are indicated by asterisks (* *p* < 0.05; *** *p* < 0.001; **** *p* < 0.0001). Abbreviations: AEA: *N*-arachidoyl-ethanolamine; 13-HODE-EA: 13-hydroxyoctadecadienoic acid ethanolamine; DHEA: *N*-docosahexaenoyl-ethanolamine; DPEA: *N*-docosapentaenoyl-ethanolamine; EPEA: *N*-eicosapentaenoyl-ethanolamine; LEA: *N*-linoleoyl-ethanolamine; MUFA: monounsaturated fatty acid; NAE: *N*-acyl-ethanolamine; PUFAs: polyunsaturated fatty acids; PS: psoriatic substitutes; PS^+EPA^: psoriatic substitutes supplemented with EPA; PS^+T^: psoriatic substitutes produced with T cells; and PS^+T+EPA^: psoriatic substitutes produced with T cells and supplemented with EPA.

The main *N*-acylethanolamines (NAEs) quantified in the epidermis of the skin substitutes were 13-hydroxyoctadecadienoic acid ethanolamine (13-HODE-EA) and *N*-linoleoyl-ethanolamine (LEA), both derivatives of n-6 PUFAs (Figure 5A–C). No impact was detected on the n-6 PUFA-NAE levels with the addition of EPA (Figure 5C). The supplementation of the culture media with EPA elevated the levels of NAEs derived from n-3 PUFAs with significant increases in *N*-eicosapentaenoyl-ethanolamine (EPEA) levels in PS^+EPA^ and PS^+T+EPA^ compared with their respective counterparts (Figure 5B). The amounts of *N*-docosahexaenoyl-ethanolamine (DHEA) were upregulated in PS^+T+EPA^ compared with healthy and psoriatic controls (Figure 5B).

## 4. Discussion

Psoriasis is a complex autoimmune disease for which the exact etiology is still unknown. In recent years, abundant experimental evidence has highlighted the important lipid dysregulation of the disease [12,24,34,35]. The predominant activation of the n-6 PUFA metabolism in psoriatic lesions also contributes to the inappropriate stimulation of the adaptive immunity in psoriatic skin, mainly through n-6 PUFA interaction with T cells. In contrast, n-3 PUFAs, which are known for their immunomodulating properties, are downregulated in psoriatic skin and dietary n-3 PUFA supplementation has been repeatedly shown to improve the symptoms of the disease [19,20]. Although the quantification of the levels of prostanoids and eicosanoids in the skin and blood of psoriatic patients is a well-established practice, the impact of n-3 PUFA enrichment, particularly EPA, on the lipidic profile of psoriatic skin is not yet known. The results of our study showed that EPA supplementation for the psoriatic skin model enriched with polarized T cells modifies the membrane phospholipid portrait of the substitutes, with a strong incorporation of EPA into the dermis and epidermis. The added EPA was also transformed into longer-chain n-3 PUFAs, which participate in the restoration of the epidermal differentiation program of psoriatic keratinocytes. Inversely, a downregulation of n-6 PUFA metabolites was observed in EPA-supplemented psoriatic skin substitutes. These effects were also transposed to the levels of n-3 and n-6-PUFA lipid mediators with increased levels of n-3-lipid derivatives in psoriatic substitutes supplemented with EPA, particularly PGE_3_, 12-HEPE, 17-HDHA and EPEA, while decreased levels of n-6-lipid derivatives such as 12-HETE and PGE_2_ were observed.

In the current study, EPA was correctly incorporated into the phospholipid fraction of the dermis and epidermis of the skin substitutes (PS^+EPA^ and PS^+T+EPA^), demonstrating the effectiveness of the culture media supplementation method. The added EPA was metabolized in both cutaneous compartments, mainly into n-3 DPA and DHA, showing that n-3 PUFA metabolism remains effective even in the presence of T cells and corresponds to the typical metabolic pathway observed in the skin [36,37]. For the first time, we demonstrate here a significant increase in DHA levels following supplementation with a n-3 PUFA other than DHA itself. When we supplemented our immunocompetent psoriatic model with ALA, it did not increase the levels of DHA while modestly increasing those of EPA and n-3 DPA, in sharp contrast with the EPA data presented here, suggesting that EPA, but not ALA, is more prone to elongation and desaturation into DHA [23]. As expected, the phospholipids of PS and PS^+T^ displayed overall higher proportions of n-6 PUFAs compared with the healthy model for both the epidermis and dermis, suggesting a predominant pro-inflammatory environment in psoriatic conditions. These results are consistent not only with data from our previous studies but also from other studies in which significant upregulation of n-6 PUFA metabolites was observed in psoriatic skin [17,22,23,24,38]. Among the different n-6 PUFAs, LA and AA were the most augmented in the psoriatic substitutes. It has been known since the 90s that AA is strongly implicated in psoriasis, participating in the activation and recruitment of inflammatory cells and cytokines [39]. However, dermal and epidermal levels of LA in PS^+T^ were diminished compared with PS, which could be explained by the production of LA-derived lipid mediators by both psoriatic keratinocytes and T cells. Despite the fact that there are currently no studies showing an important production of LA-lipid mediators by T cells, it is known that macrophages can synthesize 13-HODE, which then amplifies their immune function [40]. The addition of EPA had essentially the same effects on the phospholipid n-6 PUFA profiles of the dermis and epidermis, mainly with decreases in AA levels but not to the same extent since the impact of EPA was only significant in the dermis. In the dermis, exogenously added EPA in both PS^+EPA^ and PS^+T+EPA^ diminished the phospholipid levels of AA, thus limiting the proportion of metabolites involved in the chronic inflammation of psoriasis. Fundamentally, this disparity observed between the cutaneous compartments could be caused by a defective desaturase activity in the epidermis resulting in poor ability to form long-chain PUFAs [41]. The addition of EPA did not change the fact that the epidermis has difficulty converting LA to AA. Inversely, the dermis displays powerful desaturase activity and provides a biochemical support to the epidermis due to the important crosstalk between the two skin layers. Therefore, the epidermis depends partly on the dermis for local production of long-chain PUFAs, which explains, in part, the lower impact of EPA on the epidermal phospholipid composition [42]. It should also be kept in mind that totally polarized T cells, particularly Th1 cells, do not easily change state in response to different microenvironments, which could influence the incorporation of EPA into the membrane phospholipids of these cells [43].

In membrane phospholipids, n-3 and n-6 PUFAs can be released and metabolized into bioactive lipid mediators, which can subsequently activate different signaling pathways [44]. These bioactive lipid mediators play important roles in the development and resolution of inflammation, and some n-6 PUFA-lipid mediators are upregulated in psoriatic skin [22,24,34]. Here, supplementation with EPA increased the epidermal levels of n-3 PUFA-lipid mediators—mainly PGE_3_, 12-HEPE and 17-HDHA—only in PS^+EPA^, and not in PS^+T+EPA^. This result suggests that the presence of T cells could block the subsequent metabolization of n-3 PUFAs into their bioactive derivatives. In vivo, a dietary supplementation with EPA resulted in the upregulation of the production of EPA-derived HEPEs in both the epidermis and the plasma of heathy humans [42,45]. On the other hand, a recent study showed that in a psoriatic context, DHA is more effective than EPA in producing anti-inflammatory and pro-resolution metabolites [46]. Although it is recognized that COX-2 action is augmented in Th cells, very little is known about the exact profile of prostanoids and eicosanoids produced by T cells, especially those derived from n-3 PUFAs [47]. The impact of n-3 PUFA prostaglandins in the skin has also been little studied [48,49]. N-3 PUFAs generally inhibit AA metabolism by competing with the latter for cyclooxygenase (COX) and lipoxygenase (LOX) enzymes [50]. The phospholipid membrane of T cells also prioritizes the incorporation of n-6 PUFAs over that of n-3 PUFAs for cell proliferation [23,51]. Finally, it should be pointed out that, in the present study, the last EPA supplementation was carried out 48 h before the samples were harvested and that most of the lipid mediators have very short half-lives, rapidly mediating their effects following their production [52]. Interestingly, supplementation with EPA in both PS^+EPA^ and PS^+T+EPA^ augmented the epidermal EPEA content, suggesting that in presence of T cells the conversion of n-3 PUFAs is performed preferentially towards NAEs. Since 2001, some studies have shown that EPA and DHA are rapidly converted into their respective NAE derivatives DHEA and EPEA in diverse tissues [53,54,55,56].

COX-2 derivatives obtained from AA were strongly detected in PS and PS^+T^, especially PGE_2_, which is representative of what is found in psoriatic skin in vivo [34]. A few studies have also reported the production of PGE_2_, PGD_2_ and its dehydration product 15-deoxy-Δ-PGJ_2_ in Th cells and Jurkat T cells [49,57,58]. Here, supplementation of the culture media with EPA decreased the epidermal levels of PGE_2_ in PS^+T+EPA^ to the benefit of PGE_3_. Over the years, numerous studies in healthy humans, murine models and disease models have shown a decreased production of PGE_2_ following supplementation with diverse n-3 PUFA metabolites [46,59,60,61,62]. In healthy humans, PGE_2_ was found to be diminished in monocytes following a diet rich in EPA and DHA [63]. We have also previously demonstrated significant reductions in PGE_2_ levels in our classic psoriatic model (without immune cells) following supplementation with either ALA or DHA [22,24]. However, we are, to our knowledge, the first group to demonstrate that EPA can reduce PGE_2_ levels in a psoriatic context with a strong inflammatory environment augmented by T cells.

Of all the mediators that we quantified, the 15-LOX metabolite 13-HODE was the most abundant lipid mediator produced in psoriatic skin substitutes, particularly in PS. This is in agreement with previous studies showing overexpression of 15-LOX in skin substitutes as well as a high production of 13-HODE in psoriatic skin [29,34]. However, the addition of EPA had no effect on 13-HODE levels. Furthermore, 12-HETE levels were higher in psoriatic substitutes than in the healthy ones, which is consistent with results found in vivo and in vitro [24,34,64]. 12-HETE has been found to be involved in T cell functions, and the upregulation of this mediator leads to the development of autoimmune diseases [47,65]. 12-HETE and LTB_4_ are chemoattractants for many leukocytes including T cells, and they both contribute to the infiltration of immune cells in psoriatic skin [66,67]. The supplementation of the culture media with EPA diminished the levels of 12-HETE and increased those of 12-HEPE in both PS^+EPA^ and PS^+T+EPA^, meaning that EPA might affect the migration of T cells in our model. These results are in line with previous studies [22,24]. In fact, in our latest studies, the addition of n-3 PUFAs restricted the T cells to the dermis of the psoriatic substitutes and limited their migration to the epidermis [23]. Overall, n-6 PUFA HFAs were more strongly detected in PS than in PS^+T^, suggesting that the addition of T cells altered the action of 12-LOX in our model. The role of 12-LOX in T cells has yet to be investigated, but some studies have shown that B cells possess a system to downregulate the activity of some LOXs [68].

## 5. Conclusions

In summary, our results demonstrated that EPA modulates the lipid profile of psoriatic substitutes towards an anti-inflammatory state. The EPA added to our psoriatic model containing T cells was incorporated into the epidermal and dermal phospholipids of the substitutes, which increased the overall levels of EPA-lipid mediators, mainly PGE_3_, 12-HEPE and EPEA. In parallel, supplementation with EPA decreased the levels of inflammatory lipid mediators derived from n-6 PUFAs, particularly PGE_2_ and 12-HETE, even in the presence of T cells. Therefore, our study suggests that EPA could counteract the inflammatory environment created by psoriatic keratinocytes and T cells and could promote the return to a steadier state. Since little is known about the lipid mediators produced directly by T cells, our study highlights the interest of studying in detail their mechanisms of action in immune cells, and their possible effect on skin diseases. The impact of lipid mediators derived from EPA on the immune component of psoriasis has yet to be revealed and could shed light on possible new therapeutic targets for this pathology.

## Data Availability

All data are contained within the manuscript or in the supplementary materials.

## Supplementary Materials

The following supporting information can be downloaded at: https://www.mdpi.com/article/10.3390/biom13091413/s1. Figure S1: Epidermal and dermal phospholipid quantification of the skin substitutes. Figure S2: n-3 and n-6-PUFA lipid mediator profile of the psoriatic skin conditions. Table S1: Levels of fatty acids in phospholipids of the epidermis of the skin substitutes after EPA supplementation. Table S2: Levels of fatty acids in phospholipids of the dermis of the skin substitutes after EPA supplementation. Table S3: Levels of bioactive lipids in the skin substitutes after EPA supplementation. Table S4: Levels of bioactive lipids in the skin substitutes produced with T cells after EPA supplementation.
