# Supplementary material for: Eicosapentaenoic Acid Influences the Lipid Profile of an In Vitro Psoriatic Skin Model Produced with T Cells
Source: Biomolecules. 2023 Sep 19;13(9):1413. doi: 10.3390/biom13091413 (PMC10526348; doi:10.3390/biom13091413)
Supplement: Supplementary file 1 [file biomolecules-13-01413-s001.zip › biomolecules-2585202-supplementary.pdf]

SUPPLEMENTARY MATERIAL

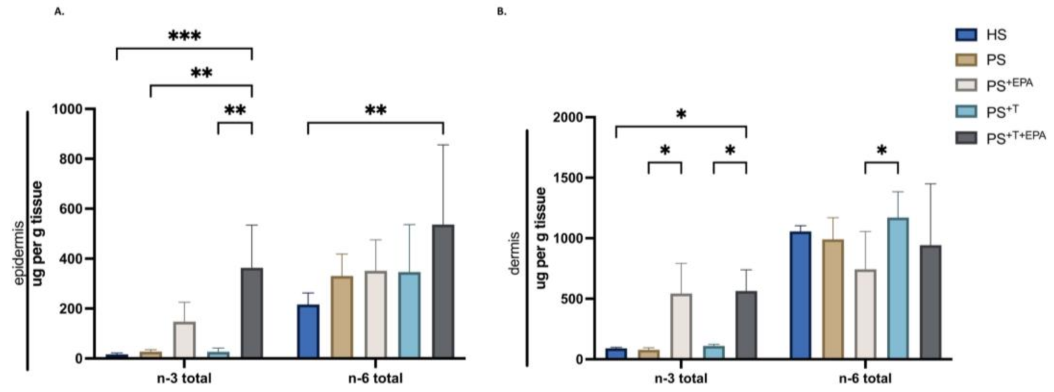

Figure S1. Epidermal and dermal phospholipid quantification of the skin substitutes. (A) Characterization of epidermal total fatty acids using gas chromatography. Impact of EPA supplementation on total n-3 and n-6 PUFAs quantities; **(B)** Characterization of dermal total fatty acids using gas chromatography. Impact of EPA supplementation on total n-3 and n-6 PUFAs quantities. The values are presented as μg/g of tissue; (N=3 donors, n=2 skin substitutes per donor). Statistical significance was determined using two-way ANOVA followed by Tukey's *post-hoc* test. Significant differences are indicated by asterisks (*p< 0.05; **p< 0.01; ***p< 0.001). Abbreviations: HS: healthy substitutes; EPA: eicosapentaenoic acid; PS: psoriatic substitutes; PS^{+EPA}: psoriatic substitutes supplemented with EPA; PS^{+T}: psoriatic substitutes produced with T cells; PS^{+T+EPA}: psoriatic substitutes produced with T cells and supplemented with EPA; T: T cells.

The comparison of the levels of lipid mediators derived from n-3 and n-6 PUFAs between the PS and PS^{+T} conditions was carried out. As for the n-3 PUFA lipid mediators, no difference between n-3 prostaglandins levels was noted between PS and PS^{+T}. As for n-3 HFAs, the amounts of 12-HEPE were decreased in PS^{+T} as compared with PS, suggesting that T cells might affect the efficiency of the 12-LOX enzyme (Figure S2.A). As for the n-6 PUFA lipid mediators, prostaglandin levels were relatively similar between PS and PS^{+T}, except for PGE_2 where a significant increase was quantified in PS^{+T}. Moreover, 12-HETE was diminished in PS^{+T} compared with PS, reinforcing the hypothesis that the addition of T cells affects the function of 12-LOX preventing it from producing both 12-HEPE and 12-HETE (Figure S2.A and S2.B).

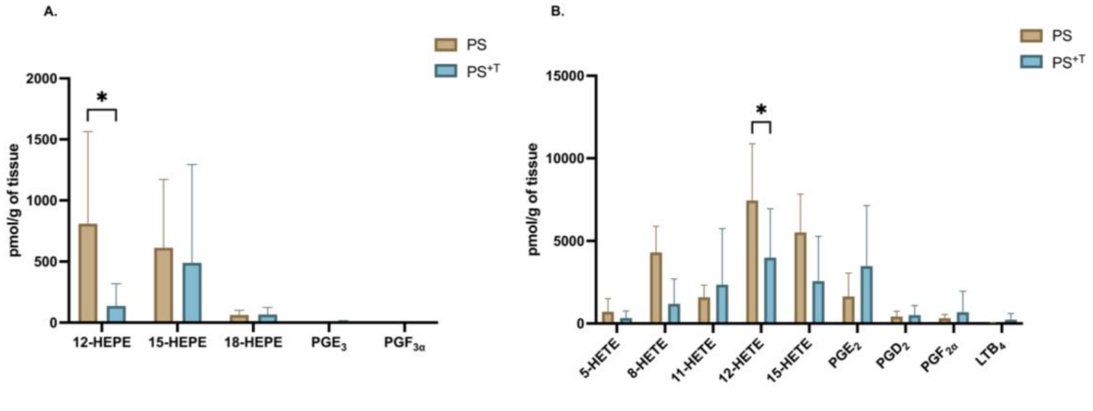

Figure S2. n-3 and n-6-PUFA lipid mediator profile of the psoriatic skin conditions. Influence of the addition of T cells to psoriatic substitutes the levels of n-3 and n-6-PUFA lipid derivatives in the epidermis of PS and PS^{+}; **(A)** Characterization of epidermal n-3 lipid mediators derived from EPA; **(B)** Characterization of epidermal n-6 lipid mediators derived from AA. (N=3 donors; n=2 skin substitutes per donor). Statistical significance was determined using two-way

ANOVA followed by Bonferroni's *post-hoc* test. Significant differences are indicated by asterisks (*p< 0.05). Abbreviations: AA: arachidonic acid; EPA: eicosapentaenoic acid; HEPE: hydroxyeicosapentaenoic acid; HETE: hydroxyeicosatetraenoic acid; T: T cells; n-3: omega-3; n-6: omega-6; PG: prostaglandin; PUFAs: polyunsaturated fatty acids; PS: psoriatic substitutes; PS^{+T}: psoriatic substitutes produced with T cells.

Supplementary tables

Table S1. Levels of fatty acids in phospholipids of the epidermis of the skin substitutes after EPA supplementation.

Epidermal phospholipids						
Mean ±SD (%)						
**Fatty acids**	**HS**	**PS**	**PS^{+EPA}**	**PS^{+T}**	**PS^{+T+EPA}**	**P-value**
**Saturated FAs**						
14:0 (Myristic acid)	1.786 ± 0.374	2.185 ± 0.216	2.235 ± 0.154	2.048 ± 0.542	1.922 ± 0.401	NS
16:0 (Palmitic acid)	14.070 ± 1.043	14.458 ± 0.483	15.625 ± 0.741	16.29 ± 1.614	16.51 ± 1.77	NS
18:0 (Stearic acid)	11.365 ± 0.783	10.852 ± 0.501	11.228 ± 0.632	11.68 ± 0.978	13.618 ± 2.74	NS
20:0	0.325 ± 0.187	0.307 ± 0.052	0.347 ± 0.06	0.405 ± 0.142	0.433 ± 0.107	NS
22:0	0.667 ± 0.301	0.510 ± 0.082	0.345 ± 0.272	0.637 ± 0.221	0.773 ± 0.299	NS
24:0	1.755 ± 0.591	1.378 ± 0.122	1.328 ± 0.101	1.678 ± 0.491	2.012 ± 0.646	NS
**n-3 PUFAs**						
t-18:3n-3	ND	ND	ND	ND	ND	NS
18:3n-3 (ALA)	ND	ND	ND	ND	ND	NS
20:3n-3 (ETE)	ND	ND	ND	ND	ND	NS
20:4n-3 (ETA)	ND	ND	ND	ND	ND	NS
20:5n-3 (EPA)	ND	0.185 ± 0.102	2.838 ± 1.201	0.082 ± 0.09	2.645 ± 0.601	<0.0001
22:3n-3	ND	ND	ND	ND	ND	NS
22:5n-3 (DPA)	0.438 ± 0.073	0.445 ± 0.069	2.942 ± 2.042	0.457 ± 0.165	4.045 ± 1.895	NS
22:6n-3 (DHA)	0.388 ± 0.054	0.437 ± 1.178	1.818 ± 62.5	0.402 ± 0.097	2.505 ± 1.294	<0.0001
**n-5 PUFAs**						
t-14:1n-5	ND	ND	ND	ND	ND	NS
14:1n-5	ND	ND	ND	ND	ND	NS
18:1n-5	0.472 ± 0.138	0.320 ± 0.0054	0.328 ± 0.067	0.437 ± 0.08	0.240 ± 0.196	NS
**n-6 PUFAs**						
18:1	ND	ND	ND	ND	ND	NS
t-18:2n-6	0.12 ± 0.134	0.357 ± 0.203	0.255 ± 0.141	0.160 ± 0.137	0.07 ± 0.109	NS
9c12t-18:2n-6	0.170 ± 0.137	0.365 ± 0.067	0.255 ± 0.051	0.257 ± 0.175	0.112 ± 0.131	NS
8t12c-18:2n-6	0.103 ± 0.161	ND	0.183 ± 0.299	0.302 ± 0.387	0.040 ± 0.098	NS
18:2n-6 (LA)	3.750 ± 0.296	5.408 ± 0.870	4.547 ± 0.866	4.332 ± 1.115	4.980 ± 0.188	0.0023
18:3n-6 (γ-linolenic acid)	0.048 ± 0.118	0.462 ± 0.276	0.252 ± 0.221	0.097 ± 0.150	0.065 ± 0.107	NS
20:2n-6	ND	0.045 ± 0.071	0.037 ± 0.058	0.042 ± 0.066	0.043 ± 0.07	NS
20:3n-6 (dihomo-γ-linolenic acid)	0.915 ± 0.256	1.255 ± 0.175	1.170 ± 0.132	1.360 ± 0.493	1.548 ± 0.434	NS
20:4n-6 (AA)	1.783 ± 1.412	3.962 ± 0.359	3.312 ± 0.461	4.030 ± 1.187	3.498 ± 0.460	<0.0001
22:2n-6	ND	ND	ND	ND	ND	NS
22:4n-6	0.623 ± 0.564	1.020 ± 0.231	0.743 ± 0.107	1.118 ± 0.329	0.903 ± 0.399	NS
22:5n-6	0.917 ± 0.123	0.195 ± 0.096	0.040 ± 0.062	0.178 ± 0.139	0.088 ± 0.110	NS
**n-7 MUFAs**						
t-16:1n-7	ND	ND	ND	ND	ND	NS
16:1n-7 (Palmitoleic acid)	8.557 ± 1.733	10.452 ± 2.072	10.355 ± 1.884	8.813 ± 2.643	6.975 ± 3.303	NS
t-18:1n-7	0.025 ± 0.061	0.05 ± 0.078	0.037 ± 0.057	0.028 ± 0.069	0.037 ± 0.057	NS
18:1n-7 (Vaccenic acid)	15.847 ± 1.809	13.723 ± 0.954	12.83 ± 0.789	13.847 ± 1.00	10.2 ± 3.807	0.0101
**n-9 MUFA**						

t-18.1n-9	0.09 ± 0.143	0.067 ± 0.103	0.052 ± 0.08	0.075 ± 0.117	0.053 ± 0.084	NS
18:1n-9 (Oleic acid)	29.92 ± 1.901	27.723 ± 2.776	27.02 ± 2.105	28.482 ± 0.76	28.88 ± 2.946	NS
20:1n-9	0.337 ± 0.263	0.408 ± 0.064	0.353 ± 0.072	0.445 ± 0.169	0.258 ± 0.203	NS
22:1n-9	ND	ND	ND	ND	ND	NS
24:1n-9	1.053 ± 0.209	0.732 ± 0.107	0.745 ± 0.176	0.992 ± 0.293	0.983 ± 0.278	NS
**n-12 MUFA**						
t-18:1n-12	ND	ND	ND	ND	ND	NS
18:1n-12	ND	ND	ND	ND	ND	NS
20:1n-12	ND	ND	ND	ND	ND	NS
**Totals**						
Total SFAs	31.323 ± 2.153	31.22 ± 0.638	32.295 ± 1.00	33.629 ± 1.129	35.937 ± 3.169	0.0003
Total PUFAs	11.852 ± 1.165	14.172 ± 1.751	15.112 ± 2.142	12.812 ± 2.65	18.535 ± 7.272	<0.0001
Total MUFAs	58.565 ± 3.241	55.338 ± 2.147	53.178 ± 2.797	54.738 ± 2.096	46.697 ± 10.117	<0.0001

Abbreviations: AA, arachidonic acid; ALA, α-linolenic acid; DHA, docosahexaenoic acid; EPA, eicosapentaenoic acid; FA, fatty acid; LA, linoleic acid; MUFA, monounsaturated fatty acid; PUFAs, polyunsaturated fatty acids; SFA, saturated fatty acid; SD, standard deviation.

Table S2. Levels of fatty acids in phospholipids of the dermis of the skin substitutes after EPA supplementation.

Dermal phospholipids						
Mean ±SD (%)						
**Fatty acids**	**HS**	**PS**	**PS^{+EPA}**	**PS^{+T}**	**PS^{+T+EPA}**	**P-value**
**Saturated FAs**						
14:0 (Myristic acid)	2.096 ± 0.161	2.145 ± 0.153	2.035 ± 0.179	2.078 ± 0.42	1.722 ± 0.398	NS
16:0 (Palmitic acid)	14.522 ± 0.607	14.557 ± 0.457	14.425 ± 0.842	15.585 ± 1.744	14.098 ± 0.817	NS
18:0 (Stearic acid)	16.632 ± 0.27	16.652 ± 0.298	17.785 ± 0.323	18.343 ± 1.253	20.047 ± 2.046	0.0012
20:0	0.182 ± 0.013	0.227 ± 0.025	0.22 ± 0.033	0.170 ± 0.096	0.237 ± 0.05	NS
22:0	0.572 ± 0.047	0.69 ± 0.072	0.7 ± 0.091	0.63 ± 0.179	0.745 ± 0.152	NS
24:0	1.15 ± 0.069	1.325 ± 0.103	1.625 ± 0.266	1.192 ± 0.186	1.582 ± 0.387	NS
**n-3 PUFAs**						
t-18:3n-3	ND	ND	ND	ND	ND	NS
18:3n-3 (ALA)	ND	ND	ND	ND	ND	NS
20:3n-3 (ETE)	ND	ND	ND	ND	ND	NS
20:4n-3 (ETA)	ND	ND	ND	ND	ND	NS
20:5n-3 (EPA)	0.050 ± 0.046	0.018 ± 0.045	3.588 ± 2.138	0.092 ± 0.156	4.077 ± 2.568	<0.0001
22:3n-3	ND	ND	ND	ND	ND	NS
22:5n-3 (DPA)	1.54 ± 0.06	1.437 ± 0.236	7.02 ± 1.599	1.688 ± 0.189	6.798 ± 1.552	<0.0001
22:6n-3 (DHA)	1.094 ± 0.109	1.158 ± 0.115	2.587 ± 1.012	1.284 ± 0.28	3.28 ± 1.323	0.0001
**n-5 PUFAs**						
t-14:1n-5	ND	ND	ND	ND	ND	NS
14:1n-5	ND	ND	ND	ND	ND	NS
18:1n-5	0.48 ± 0.051	0.357 ± 0.065	0.328 ± 0.079	0.347 ± 0.269	0.257 ± 0.158	NS
**n-6 PUFAs**						
18:1	ND	ND	ND	ND	ND	NS
t-18:2n-6	0.054 ± 0.05	ND	ND	0.012 ± 0.029	ND	NS
9c12t-18:2n-6	0.12 ± 0.019	0.092 ± 0.073	0.023 ± 0.057	0.045 ± 0.07	0.068 ± 0.075	NS
8t12c-18:2n-6	0.088 ± 0.082	0.065 ± 0.102	ND	0.022 ± 0.053	ND	NS
18:2n-6 (LA)	2.554 ± 0.222	4.642 ± 0.604	4.583 ± 0.692	3.362 ± 0.186	4.642 ± 0.692	0.0096
18:3n-6 (γ-linolenic acid)	0.136 ± 0.026	0.125 ± 0.101	0.058 ± 0.092	0.062 ± 0.068	0.078 ± 0.092	NS
20:2n-6	0.164 ± 0.032	0.238 ± 0.044	0.182 ± 0.1	0.183 ± 0.144	0.148 ± 0.132	NS

20:3n-6 (dihomo-γ-linolenic acid)	0.414 ± 0.053	0.54 ± 0.061	0.592 ± 0.09	0.455 ± 0.036	0.663 ± 0.122	NS
20:4n-6 (AA)	14.152 ± 1.688	20.395 ± 0.523	14.813 ± 2.657	19.715 ± 1.163	14.162 ± 3.66	<0.0001
22:2n-6	ND	ND	ND	ND	ND	NS
22:4n-6	2.992 ± 1.256	5.53 ± 0.627	2.223 ± 0.994	5.55 ± 0.748	1.957 ± 1.212	<0.0001
22:5n-6	0.952 ± 0.447	1.575 ± 0.283	0.363 ± 0.246	1.815 ± 0.361	0.272 ± 0.3	NS
**n-7 MUFAs**						
t-16:1n-7	ND	ND	ND	ND	ND	NS
16:1n-7 (Palmitoleic acid)	2.438 ± 0.326	2.707 ± 0.661	2.655 ± 0.923	1.968 ± 0.282	1.758 ± 0.485	NS
t-18:1n-7	0.112 ± 0.033	0.133 ± 0.066	0.087 ± 0.096	0.04 ± 0.063	0.097 ± 0.077	NS
18:1n-7 (Vaccenic acid)	7.516 ± 0.917	6.635 ± 0.627	5.485 ± 0.576	6.357 ± 0.856	4.54 ± 1.435	0.0006
**n-9 MUFA**						
t-18.1n-9	0.196 ± 0.038	0.185 ± 0.023	0.062 ± 0.098	0.123 ± 0.096	0.087 ± 0.1	NS
18:1n-9 (Oleic acid)	17.702 ± 0.793	16.363 ± 0.557	16.08 ± 0.439	16.802 ± 0.793	16.825 ± 1.064	0.0462
20:1n-9	0.474 ± 0.072	0.363 ± 0.074	0.285 ± 0.084	0.317 ± 0.170	0.178 ± 0.169	NS
22:1n-9	ND	ND	ND	ND	ND	NS
24:1n-9	2.01 ± 0.071	1.858 ± 0.183	1.74 ± 0.351	1.748 ± 0.228	1.518 ± 0.37	NS
**n-12 MUFA**						
t-18:1n-12	ND	ND	ND	ND	ND	NS
18:1n-12	ND	ND	ND	ND	ND	NS
20:1n-12	ND	ND	ND	ND	ND	NS
**Totals**						
Total SFAs	36.38 ± 0.432	36.817 ± 0.542	37.862 ± 1.124	38.805 ± 2.295	39.345 ± 1.756	0.0003
Total PUFAs	33.822 ± 0.826	35.808 ± 0.883	36.273 ± 1.62	34.303 ± 2.065	36.153 ± 2.245	0.0004
Total MUFAs	32.424 ± 1.284	30.068 ± 1.129	27.725 ± 1.216	28.778 ± 1.498	26.018 ± 2.688	<0.0001

Abbreviations: AA, arachidonic acid; ALA, α-linolenic acid; DHA, docosahexaenoic acid; EPA, eicosapentaenoic acid; FA, fatty acid; LA, linoleic acid; MUFA, monounsaturated fatty acid; PUFAs, polyunsaturated fatty acids; SFA, saturated fatty acid; SD, standard deviation.

Table S3. Levels of bioactive lipids in the skin substitutes after EPA supplementation.

	Mean ±SD (pmol/g of tissue)			P-value	
**Bioactive lipids**	**HS**	**PS**	**PS^{+EPA}**	**PS vs PS^{+EPA}**	**PS vs HS**
**EPA derivatives**					
PGE_3	0.853 ± 2.09	ND	360.83 ± 442.99	0.0191	NS
$PGF_{3\alpha}$	1.227 ± 3.005	ND	ND	NS	NS
12-HEPE	210.65 ± 278.51	809.65 ± 754.32	3721.27 ± 3011.2	0.0038	NS
15-HEPE	363.66 ± 763.37	612.63 ± 560.14	2169.45 ± 2097.3	NS	NS
18-HEPE	22.17 ± 10.35	62.38 ± 38.06	668.49 ± 326.43	NS	NS
LTB_5	ND	ND	ND	NS	NS
**DHA derivatives**					
14-HDHA	887.45 ± 555.26	835.71 ± 736.33	1219.96 ± 969.95	NS	NS
17-HDHA	1142.95 ± 1132.65	2475.9 ± 1809.7	10561.7 ± 9171.6	0.0008	NS
RvD_5	ND	27.9 ± 36.4	1.22 ± 2.45	NS	NS
Maresin 2	2.43 ± 3.77	ND	ND	NS	NS
**LA derivatives**					
9-HODE	7347.5 ± 3457.5	25210.8 ± 11934.1	12532.7 ± 14265.4	NS	NS
13-HODE	28383.5 ± 21301.3	148139.3 ± 141509.9	55890.9 ± 34010.8	NS	0.0175
**DGLA derivatives**					
PGE_1	8.32 ± 20.37	25.6 ± 51.3	80.1 ± 46.5	NS	NS
6-keto $PGF_{1\alpha}$	12.5 ± 15.9	4.8 ± 5.6	5.4 ± 6.2	NS	NS
15-HETrE	72.3 ± 57.04	334.9 ± 179.2	113.8 ± 24.6	<0.0001	<0.0001
LTB_3	ND	ND	ND	NS	NS

**AA derivatives**					
PGE_2	134.37 ± 52.9	1638.1 ± 1408.4	488.3 ± 330.6	NS	NS
PGD_2	39.9 ± 66.1	422.5 ± 325.3	250.8 ± 427.1	NS	NS
1a1b-dihomo $PGF_{2\alpha}$	4.1 ± 1.79	6.83 ± 2.7	2.0 ± 2.35	NS	NS
$PGF_{2\alpha}$	11.9 ± 13.5	319.7 ± 219.6	599.3 ± 814	NS	NS
5-HETE	630.9 ± 249.1	706.2 ± 805.4	302.9 ± 261.6	NS	NS
8-HETE	1004.1 ± 559.8	4300.6 ± 1587.2	801.8 ± 528.1	0.0284	0.0286
11-HETE	626.1 ± 209.9	1586.3 ± 733.9	702.7 ± 199.2	NS	NS
12-HETE	899.4 ± 510.8	7446.8 ± 3428.6	1885.2 ± 945.4	<0.0001	<0.0001
15-HETE	644.9 ± 485.2	5516.7 ± 2306.3	2173.8 ± 2167.3	0.0251	0.0002
LTB_4	17.57 ± 16.7	211.5 ± 381.9	194.4 ± 393.3	NS	NS

Abbreviations: AA, arachidonic acid; ALA, α-linolenic acid; DGLA, dihomo-gamma-linolenic acid; DHA, docosahexaenoic acid; EPA, eicosapentaenoic acid; HEPE, hydroxyeicosapentaenoic acid; HETE, hydroperoxyeicosatetraenoic acid; HETrE, hydroxyeicosatrienoic acid; HDHA, hydroxydocosahexaenoic acid; HODE, hydroxyoctadecadienoic acid; LA, linoleic acid; LT, Leukotriene; N.D., not detected; PG, prostaglandin; Rv, Resolvin; SD, standard deviation.

Table S4. Levels of bioactive lipids in the skin substitutes produced with T cells after EPA supplementation.

	**Mean ±SD (pmol/g of tissue)**		**P-value**
**Bioactive lipids**	**PS^{+T}**	**PS^{+T+EPA}**	**PS^{+T} vs PS^{+TEPA}**
**EPA derivatives**			
PGE_3	6.67 ± 8.967	293.32 ± 405.06	NS
$PGF_{3\alpha}$	ND	22.82 ± 55.90	NS
12-HEPE	137.27 ± 181.53	1665.95 ± 2344.6	NS
15-HEPE	489.5 ± 803.55	1532.95 ± 2350.13	NS
18-HEPE	65.65 ± 58.58	538.77 ± 203.93	NS
LTB_5	ND	ND	NS
**DHA derivatives**			
14-HDHA	151.72 ± 211.34	410.99 ± 549.47	NS
17-HDHA	2030.9 ± 2271.9	4284.2 ± 3994.6	NS
RvD_5	ND	ND	NS
Maresin 2	ND	ND	NS
**LA derivatives**			
9-HODE	13521.7 ± 16007.7	8134.3 ± 10863.7	NS
13-HODE	87296 ± 132641.6	44923.6 ± 45115.6	NS
**DGLA derivatives**			
PGE_1	40.9 ± 58.4	80.3 ± 81.7	NS
6-keto $PGF_{1\alpha}$	32.05 ± 48.9	2.4 ± 4.9	NS
15-HETrE	138.8 ± 160.1	77.3 ± 45.8	NS
LTB_3	6.9 ± 17.1	ND	NS
**AA derivatives**			
PGE_2	2813.3 ± 3500.4	911.2 ± 990.4	0.0085
PGD_2	507.9 ± 577.7	219.3 ± 490.4	NS
1a1b-dihomo $PGF_{2\alpha}$	6.67 ± 2.6	0.79 ± 1.59	NS
$PGF_{2\alpha}$	677.2 ± 1274.9	396.7 ± 766.3	NS
5-HETE	327.1 ± 427.3	130.9 ± 153.4	NS
8-HETE	1188.5 ± 1510.2	1525.3 ± 3026.7	NS
11-HETE	2355.1 ± 3387.2	448.9 ± 364.1	NS
12-HETE	3986.5 ± 2955.5	411.9 ± 301.1	0.0232
15-HETE	2567.8 ± 2701.5	1619.6 ± 2035.1	NS
LTB_4	225.1 ± 373.5	113.3 ± 235.6	NS

Abbreviations: AA, arachidonic acid; ALA, α-linolenic acid; DGLA, dihomo-gamma-linolenic acid; DHA, docosahexaenoic acid; EPA, eicosapentaenoic acid; HEPE, hydroxyeicosapentaenoic acid; HETE, hydroperoxyeicosatetraenoic acid; HETrE, hydroxyeicosatrienoic acid; HDHA, hydroxydocosahexaenoic acid; HODE, hydroxyoctadecadienoic acid; LA, linoleic acid; LT, Leukotriene; N.D., not detected; PG, prostaglandin; Rv, Resolvin; SD, standard deviation.
